# Supplementary material for: Hazardous Heavy Metals Accumulation and Health Risk Assessment of Different Vegetable Species in Contaminated Soils from a Typical Mining City, Central China
Source: Int J Environ Res Public Health. 2021 Mar 5;18(5):2617. doi: 10.3390/ijerph18052617 (PMC7967305; doi:10.3390/ijerph18052617)
Supplement: Supplementary file 1 [file ijerph-18-02617-s001.pdf]

Hazardous Heavy Metals Accumulation and Health Risk Assessment of Different Vegetable Species in Contaminated Soils from a Typical Mining City, Central China

Zhen Wang [1,2], Jianguo Bao [1,*], Tong Wang [1], Haseeb Tufail Moryani [1], Wei Kang [2,*], Jin Zheng [2], Changlin Zhan [2] and Wensheng Xiao [2]

[1] School of Environmental Studies, China University of Geosciences, Wuhan 430074, China; wz5781@126.com (Z.W.); wangtong_1013@126.com (T.W.); haseebmoriani@cug.edu.cn (H.T.M.)
[2] School of Environmental Science and Engineering, Hubei Key Laboratory of Mine Environment Pollution Control and Remediation, Hubei Polytechnic University, Huangshi 435003, China; zhengjin0202@126.com (J.Z.); chl_zhan@126.com (C.Z.); wsxiao2002@126.com (W.X.)
* Correspondence: bjianguo@cug.edu.cn (J.B.); kangwei@hbpu.edu.cn (W.K.)

Supplementary Materials

List

1. Table S1: Results of single-factor index and Nemerow´s synthetical pollution index.
2. Table S2: Concentration (mg/kg, Fresh weight) of heavy metals in the foodstuffs of the four categories of vegetable gardens.
3. Table S3: The assessment criteria in edible parts of different vegetables (mg/kg).
4. Table S4: Principal component analysis for heavy metals of soils.
5. Table S5: Indices of non-carcinogenic risk for children.
6. Figure S1: Potential ecological risk index (RI) of single element in the four zones: (a) Non-ferrous metals smelter; (b) Abandoned copper mine; (c) Limestone quarry; (d) Iron mine.
7. Figure S2: Spatial distribution pollution levels of Igeo-Cu in the four zones: (a) Non-ferrous metals smelter; (b) Abandoned copper mine; (c) Limestone quarry; (d) Iron mine.
8. Figure S3: Spatial distribution pollution levels of Igeo-Cr in the four zones: (a) Non-ferrous metals smelter; (b) Abandoned copper mine; (c) Limestone quarry; (d) Iron mine.
9. Figure S4: Spatial distribution pollution levels of Igeo-Pb in the four zones: (a) Non-ferrous metals smelter; (b) Abandoned copper mine; (c) Limestone quarry; (d) Iron mine.
10. Figure S5: Spatial distribution pollution levels of Igeo-As in the four zones: (a) Non-ferrous metals smelter; (b) Abandoned copper mine; (c) Limestone quarry; (d) Iron mine.
11. Figure S6: Correlation coefficients of heavy metals between soils and foodstuffs: (a) Non-ferrous metals smelter; (b) Abandoned copper mine; (c) Limestone quarry; (d) Iron mine..
12. Figure S7: Principal component analysis for heavy metals of soils in the four zones: (a) Non-ferrous metals smelter; (b) Abandoned copper mine; (c) Limestone quarry; (d) Iron mine..
13. Figure S8: Cluster analysis (CA) results for five heavy metals of soils: (a) Non-ferrous metals smelter; (b) Abandoned copper mine; (c) Limestone quarry; (d) Iron mine.
14. Figure S9: The health risk contribution rates of five different elements in the four zones: (a) Non-ferrous metals smelter; (b) Abandoned copper mine; (c) Limestone quarry; (d) Iron mine..

Table S1. Results of single-factor index and Nemerow´s synthetical pollution index.

Sampling Areas		P_{Cu}	P_{Cr}	P_{Pb}	P_{Cd}	P_{As}	P_n	Class
	Min	1.16	0.31	0.23	4.33	2.52	3.24	
Non-ferrous metals smelter (*n* = 21)	Max	8.59	0.97	1.83	25.05	0.15	18.44	V
	Mean	4.75	0.39	1.14	12.98	1.42	9.64	
	Min	3.26	0.33	0.28	0.50	0.10	2.49	
Abandonted copper mine (*n* = 26)	Max	24.64	0.88	2.48	10.94	3.97	17.95	IV～V
	Mean	12.25	0.59	1.12	4.48	1.29	9.40	
	Min	0.75	0.03	0.16	0.66	0.12	0.67	
Limestone quarry (*n* = 20)	Max	1.24	0.50	0.61	3.44	1.84	2.60	II～IV
	Mean	0.94	0.28	0.36	1.84	1.03	1.46	
	Min	0.40	0.33	0.52	0.16	0.00	0.56	
Iron mine (*n* = 23)	Max	1.29	0.54	1.09	2.50	0.98	1.87	I～III
	Mean	0.73	0.39	0.75	1.17	0.16	1.06	

Table S2. Concentration (mg/kg, Fresh weight) of heavy metals in the foodstuffs of the four categories of vegetable gardens.

Elements	Non-Ferrous Metals Smelter (A Zone)	Abandoned Copper Mine (B Zone)	Limestone Quarry (C Zone)	Iron Mine (D Zone)
Cu	2.32 ± 2.06b	4.05 ± 1.96a	* 1.50 ± 1.54b	1.28 ± 0.78b
Cr	* 0.08 ± 0.09b	0.13 ± 0.08ab	0.22 ± 0.21a	0.08 ± 0.06b
Pb	* 0.41 ± 0.45a	* 0.17 ± 0.25b	* 0.19 ± 0.19ab	0.08 ± 0.06b
Cd	* 0.15 ± 0.16a	0.14 ± 0.13a	0.12 ± 0.08a	0.05 ± 0.04b
As	0.22 ± 0.19a	0.26 ± 0.22a	* 0.18 ± 0.18a	0.03 ± 0.02b

Fresh weight can be transformed by water percent and dry weight. The value shown is mean ± S.D. * coefficients of variation (CV) ≥ 1. The different small letters in the same row stand for statistical significance at $p < 0.05$.

Table S3. The assessment criteria in edible parts of different vegetables (mg/kg, Fresh weight).

NO.	Types	Cu	Cr	Pb	Cd	As
1	Solanaceous fruits	10	0.5	0.1	0.05	0.5
2	Leafy vegetables	10	0.5	0.3	0.2	0.5
3	Nuts	10	(0.5)	0.2	0.5	(0.5)
4	Rhizome geophytes	10	(0.5)	0.2	0.1	0.5
5	Cereals	10	1.0	0.2	0.1	0.5
6	Legumes	10	1.0	0.2	0.2	-

The threshold value of vegetables would refer to GB2762-2017 for Pb, Cd, As, Cr, GB13106-91 for Cu (10 mg/kg).

Table S4. Principal component analysis for heavy metals of soils.

Sampling Areas	Components	Initial Eigenvalues		
		Total	Percentage of Variance/%	Cumulative/%
	1	3.48	69.70	69.70
	2	1.11	22.13	91.83
Non-ferrous metals smelter (A zone)	3	0.21	4.21	96.04
	4	0.12	2.48	98.51
	5	0.07	1.49	100.00
	1	2.46	49.13	49.13
	2	1.16	23.14	72.28
Abandoned copper mine (B zone)	3	0.83	16.58	88.85
	4	0.36	7.25	96.10
	5	0.20	3.90	100.00
	1	2.27	45.50	45.50
	2	1.49	29.73	75.23
Limestone quarry (C zone)	3	0.59	11.71	86.94
	4	0.41	8.18	95.12

	5	0.24	4.88	100.00
Iron mine (D zone)	1	2.18	43.67	43.67
	2	1.38	27.52	71.19
	3	0.84	16.79	87.99
	4	0.51	10.22	98.20
	5	0.09	1.80	100.00

Table S5. Indices of non-carcinogenic risk for children.

Indices		Non-Ferrous Metals Smelter (A Zone)	Abandoned Copper Mine (B Zone)	Limestone Quarry (C Zone)	Iron Mine (D Zone)
THQ	Cu	0.29	0.51	0.19	0.16
	Cr	0.13	0.22	0.37	0.13
	Pb	0.59	0.24	0.27	0.11
	Cd	0.75	0.70	0.60	0.25
	As	3.69	4.36	3.02	0.50
HI		5.45	6.03	4.45	1.15

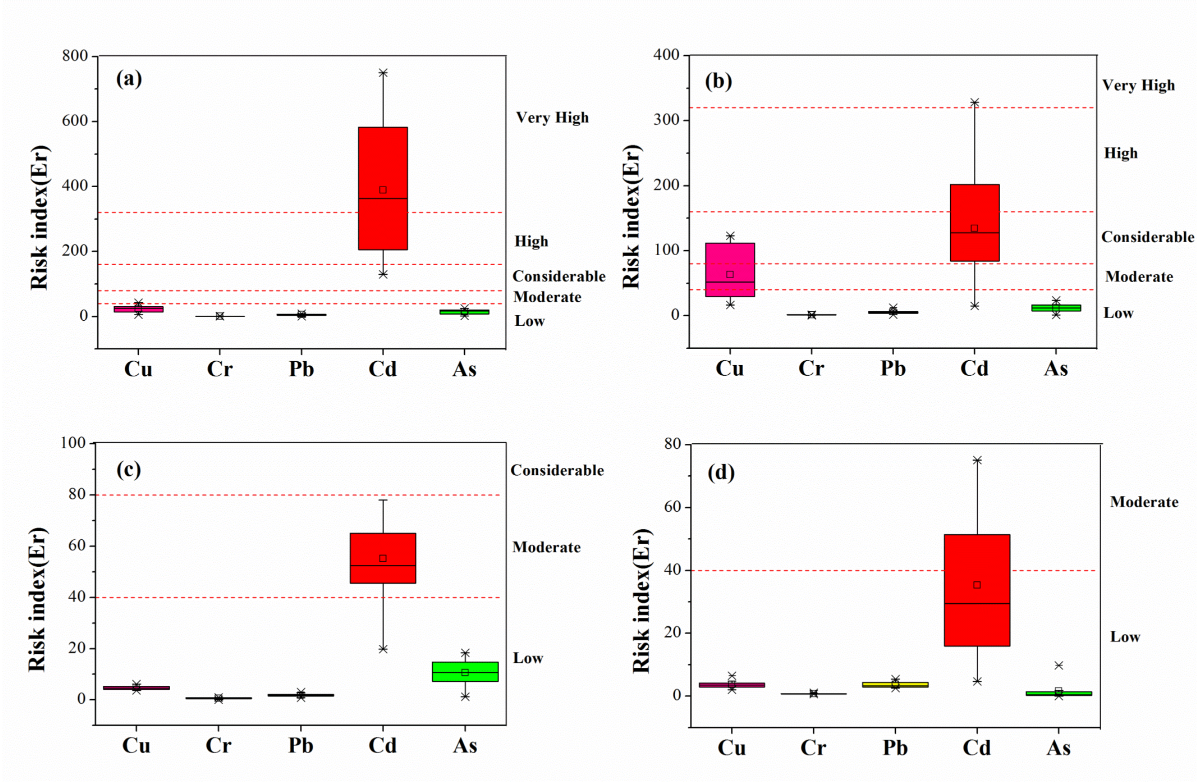

Figure S1. Potential ecological risk index (RI) of single element in the four zones: (**a**) Non-ferrous metals smelter; (**b**) Abandoned copper mine; (**c**) Limestone quarry; (**d**) Iron mine.

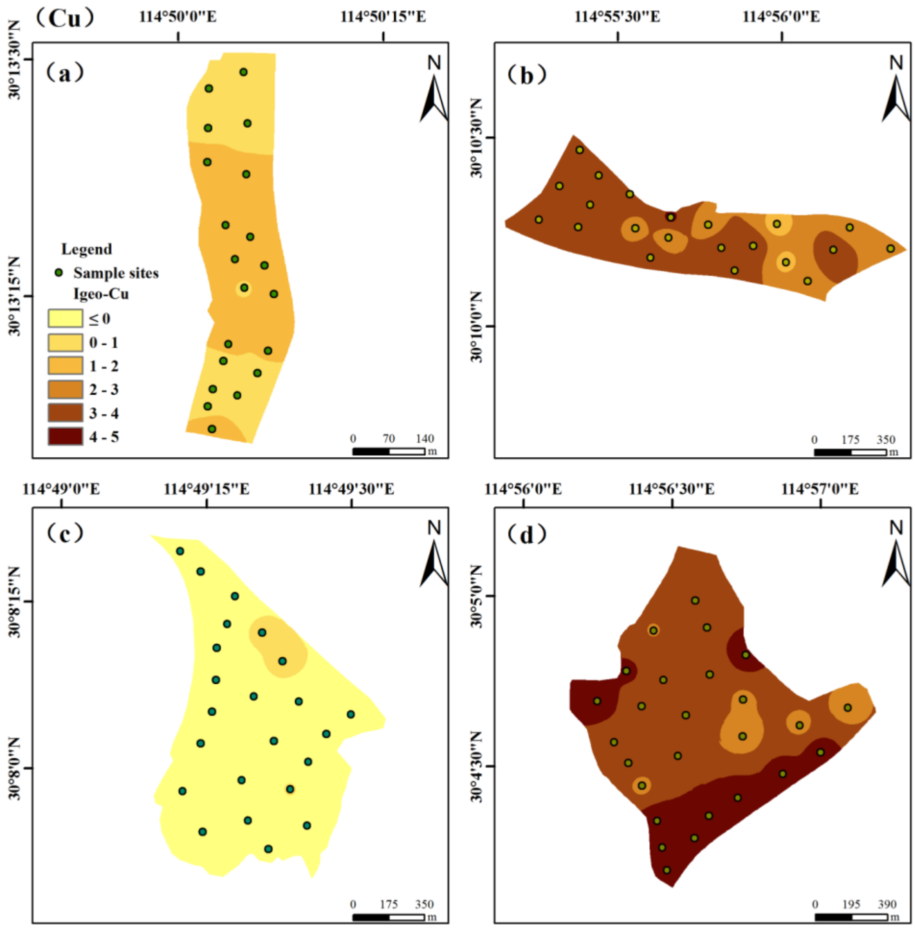

Figure S2. Spatial distribution pollution levels of Igeo-Cu in the four zones: (**a**) Non-ferrous metals smelter; (**b**) Abandoned copper mine; (**c**) Limestone quarry; (**d**) Iron mine.

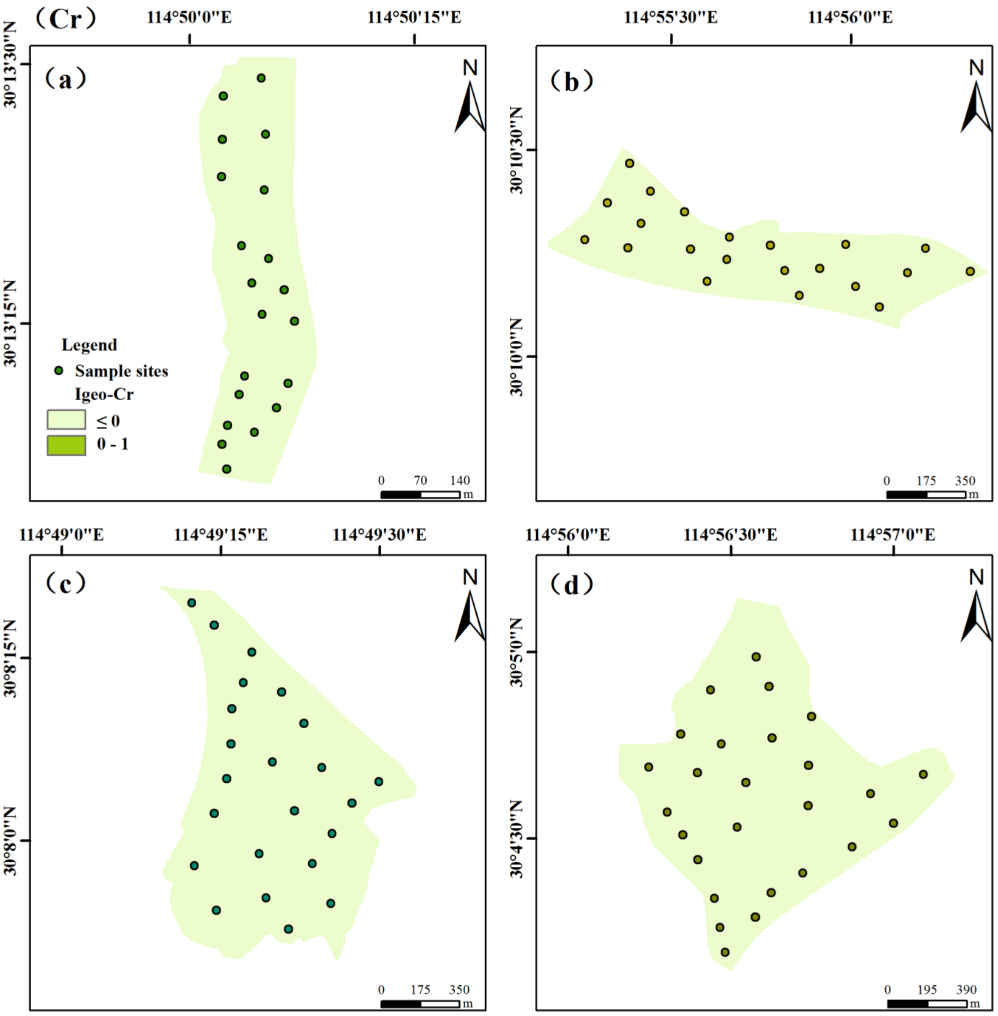

Figure S3. Spatial distribution pollution levels of Igeo-Cr in the four zones: (**a**) Non-ferrous metals smelter; (**b**) Abandoned copper mine; (**c**) Limestone quarry; (**d**) Iron mine.

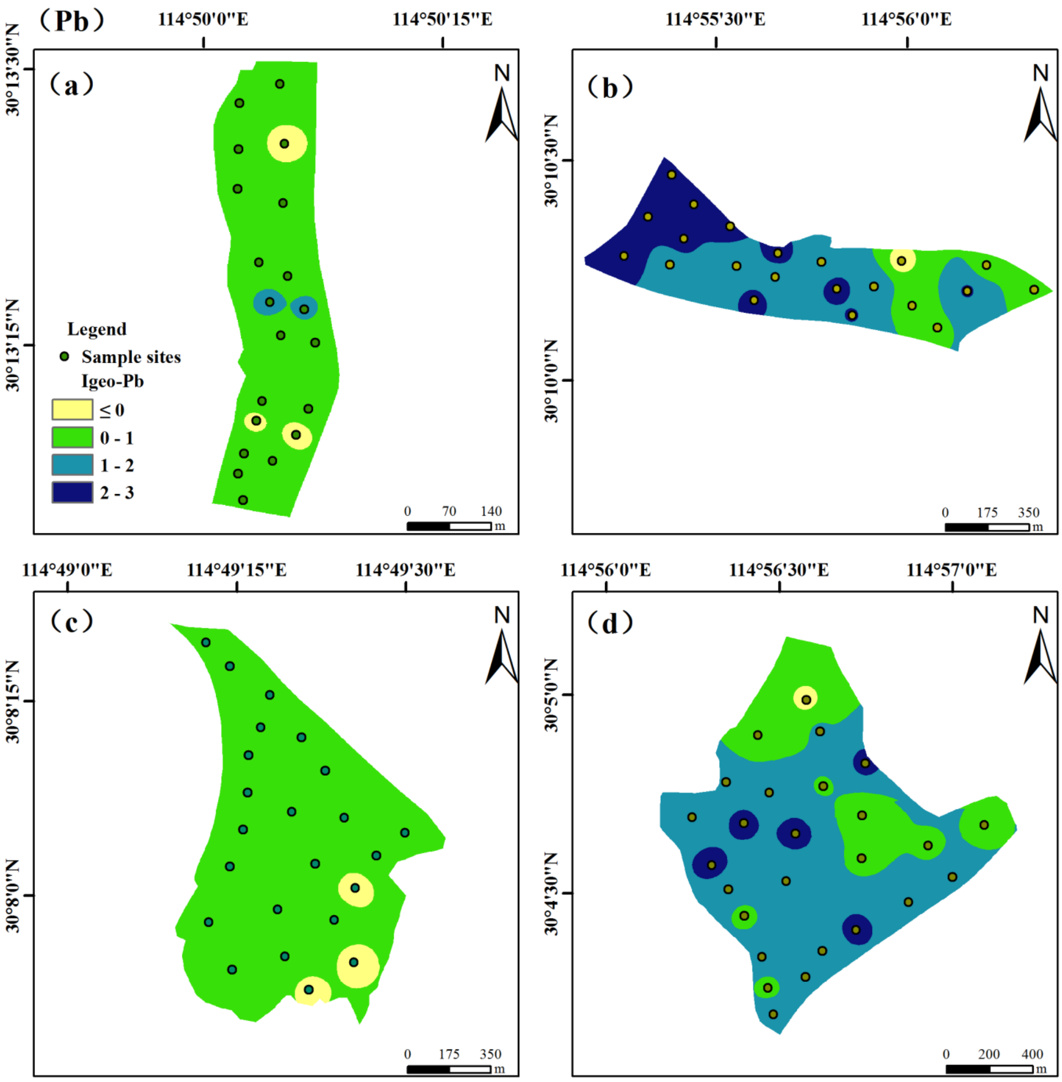

Figure S4. Spatial distribution pollution levels of Igeo-Pb in the four zones: (**a**) Non-ferrous metals smelter; (**b**) Abandoned copper mine; (**c**) Limestone quarry; (**d**) Iron mine.

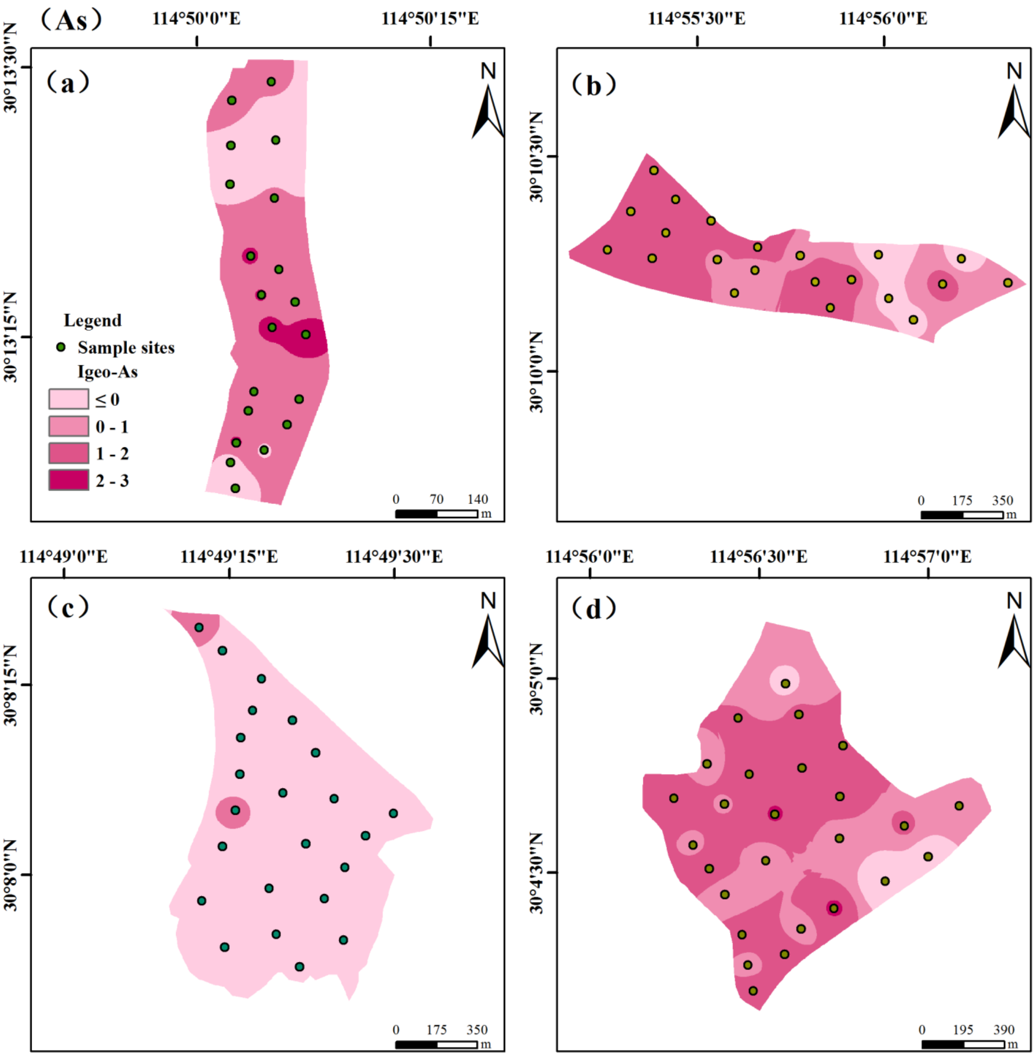

Figure S5. Spatial distribution pollution levels of Igeo-As in the four zones: (**a**) Non-ferrous metals smelter; (**b**) Abandoned copper mine; (**c**) Limestone quarry; (**d**) Iron mine.

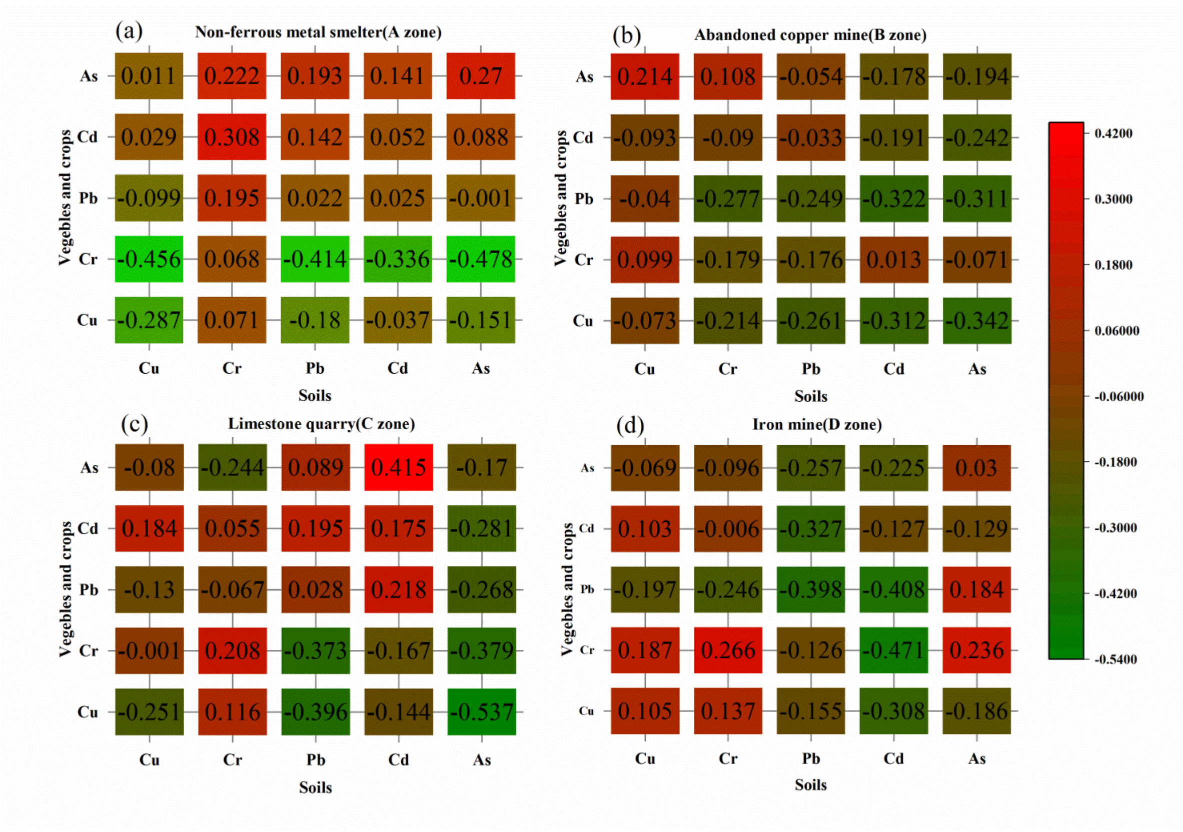

Figure S6. Correlation coefficients of heavy metals between soils and foodstuffs: (**a**) Non-ferrous metals smelter; (**b**) Abandoned copper mine; (**c**) Limestone quarry; (**d**) Iron mine.

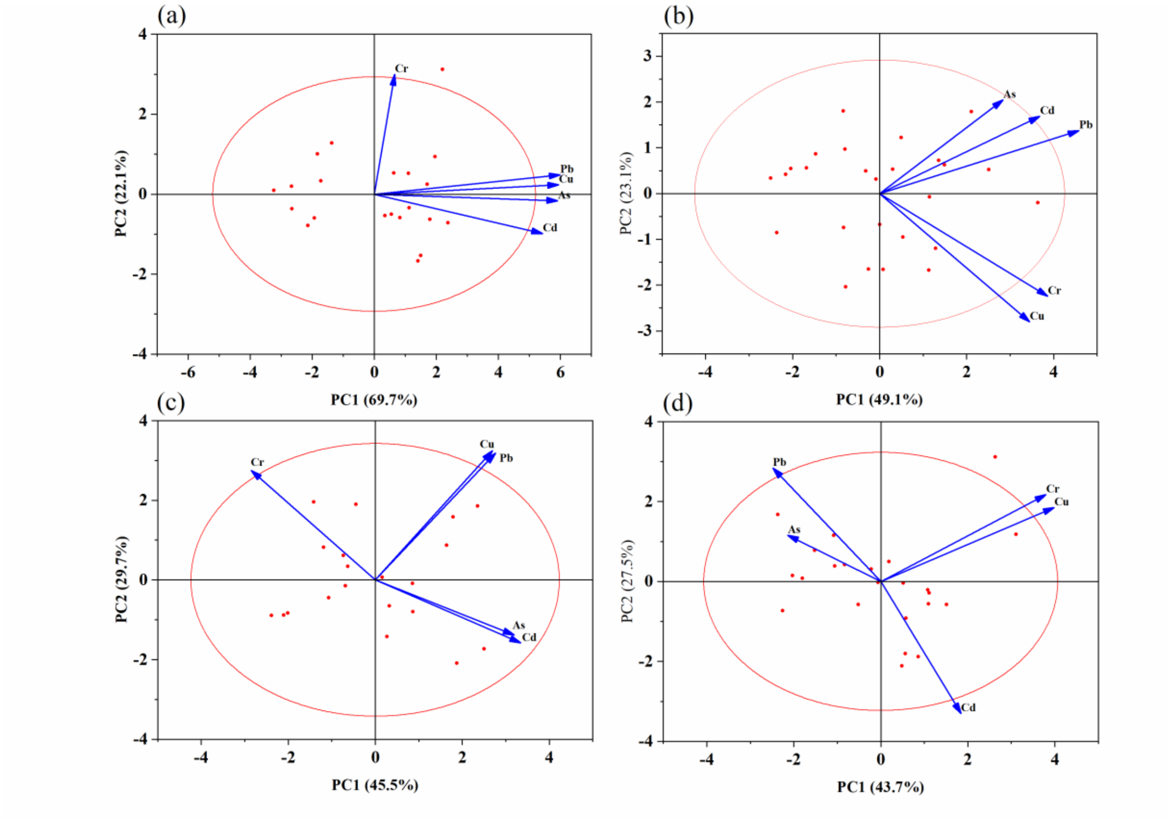

Figure S7. Principal component analysis for heavy metals of soils in the four zones: (**a**) Non-ferrous metals smelter; (**b**) Abandoned copper mine; (**c**) Limestone quarry; (**d**) Iron mine.

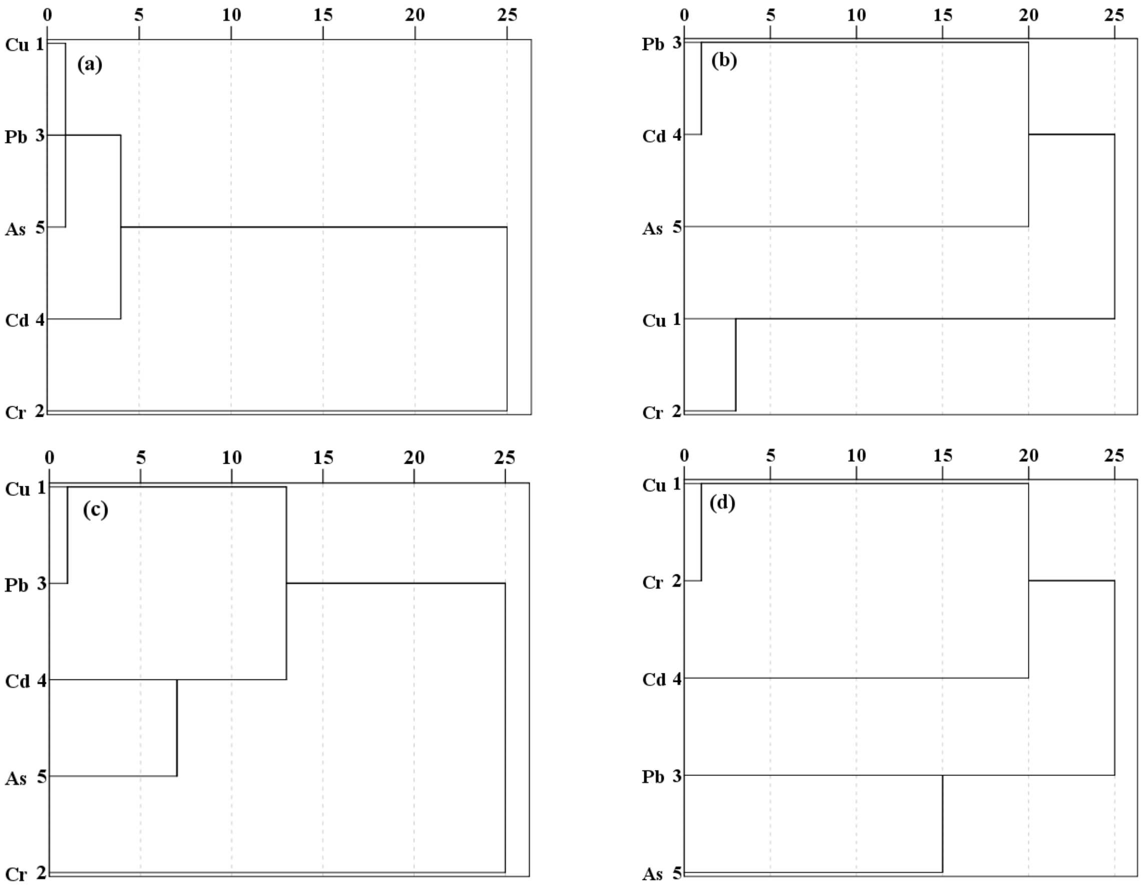

Figure S8. Cluster analysis (CA) results for five heavy metals of soils: (**a**) Non-ferrous metals smelter; (**b**) Abandoned copper mine; (**c**) Limestone quarry; (**d**) Iron mine.

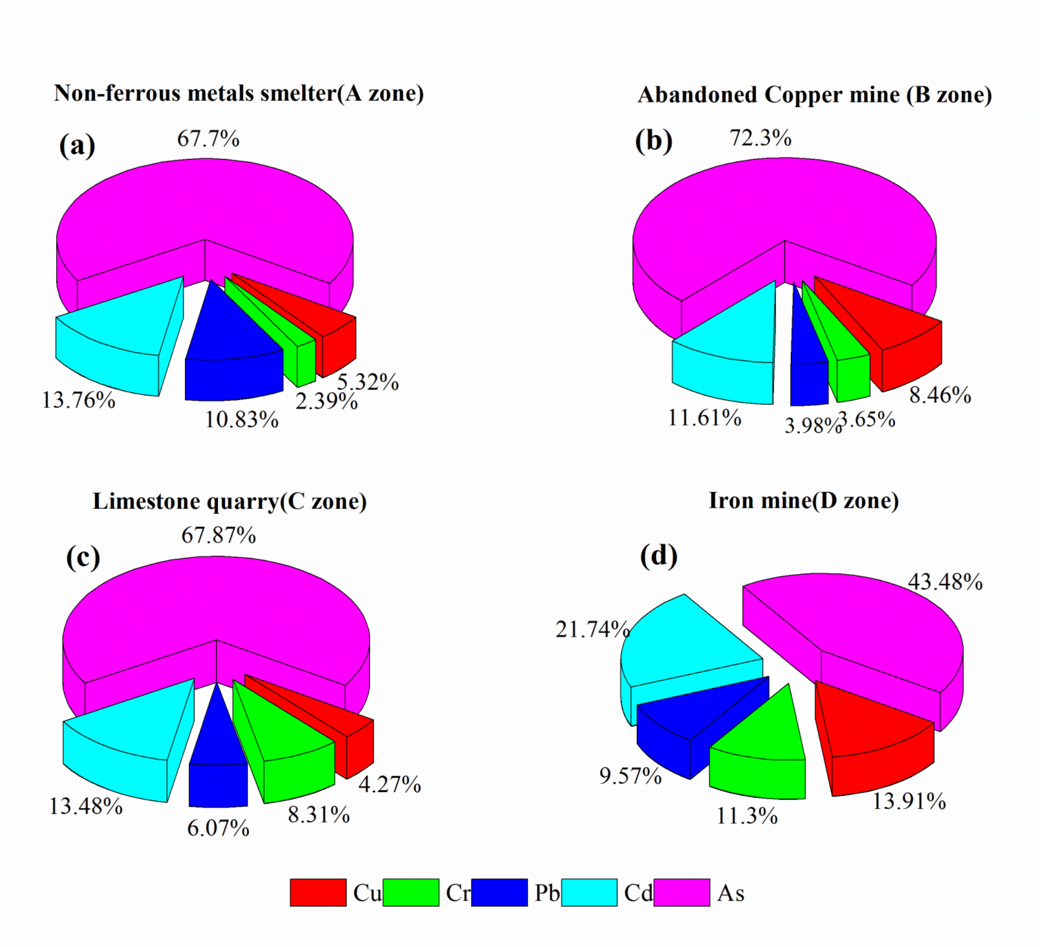

Figure S9. The health risk contribution rates of five different elements in the four zones: (**a**) Non-ferrous metals smelter; (**b**) Abandoned copper mine; (**c**) Limestone quarry; (**d**) Iron mine.
